# Prognostic impact of the expression of putative cancer stem cell markers CD133, CD166, CD44s, EpCAM, and ALDH1 in colorectal cancer

**DOI:** 10.1038/sj.bjc.6605762

**Published:** 2010-07-06

**Authors:** A Lugli, G Iezzi, I Hostettler, M G Muraro, V Mele, L Tornillo, V Carafa, G Spagnoli, L Terracciano, I Zlobec

**Affiliations:** 1 Institute of Pathology, University of Basel, Basel, Switzerland; 2 Institute for Surgical Research and Hospital Management, University of Basel, Basel, Switzerland

**Keywords:** colorectal cancer, cancer stem cell, CD44, CD166, CD133, EpCAM

## Abstract

**Background:**

The aim of this study was to elucidate the prognostic impact of putative cancer stem cell markers CD133, CD166, CD44s, EpCAM, and aldehyde dehydrogenase-1 (ALDH1) in colorectal cancer.

**Methods:**

A tissue microarray of 1420 primary colorectal cancers and 57 normal mucosa samples was immunostained for CD133, CD166, CD44s, EpCAM, and ALDH1 in addition to 101 corresponding whole tissue sections. Invasive potential of three colorectal cancer cell lines was tested.

**Results:**

Differences between normal tissue and cancer were observed for all markers (*P*<0.001). Loss of membranous CD166 and CD44s were linked to higher pT (*P*=0.002, *P*=0.014), pN (*P*=0.004, *P*=0.002), an infiltrating growth pattern (*P*<0.001, *P*=0.002), and worse survival (*P*=0.015, *P*=0.019) in univariate analysis only. Loss of membranous EpCAM expression was also linked to higher pN (*P*=0.023) and infiltrating growth pattern (*P*=0.005). The CD44s, CD166, and EpCAM expression were lost towards the invasive front. The CD44−/CD166− cells from three colorectal cancer cell lines exhibited significantly higher invasive potential *in vitro* than their positive counterparts.

**Conclusions:**

Loss, rather than overexpression, of membranous CD44s, CD166, and EpCAM is linked to tumour progression. This supports the notion that the membranous evaluation of these proteins assessed by immunohistochemistry may be representative of their cell adhesion rather than their intra-cellular functions.

Increasing evidence suggests that, similar to normal tissues, cancers might also be hierarchically organised. Only a minority of tumour cells, endowed with stem cell-like features, and thus termed cancer stem cells (CSCs), might be responsible for tumour initiation and maintenance (Reya *et al*, 2001; Pardal *et al*, 2003; Dalerba *et al*, 2007a; Visvader and Lindeman, 2008). Notably, owing to their high expression of DNA repair mechanisms, detoxifying enzymes, such as aldehyde dehydrogenase-1 (ALDH1), and molecular pumps, CSCs might survive radiochemotherapies; thus, possibly causing local recurrences and metastasis formation despite treatment (Dean *et al*, 2005; Dalerba *et al*, 2007a; Zhou *et al*, 2009).

Putative CSC populations have been identified in several types of solid tumours, on the basis of the expression of specific markers and on functional stem cell-like properties, including high clonogenicity, differentiation capacity, spheroid formation, and, critically, the ability to reproduce the original tumour on transplantation in immunodeficient mice (Dalerba *et al*, 2007a; Visvader and Lindeman, 2008).

Phenotypic characterisation of CSCs derived from colorectal cancers is still debated. While initial works identified CD133 molecule as a reliable CSC marker in primary human colorectal cancers (O’Brien *et al*, 2007; Ricci-Vitiani *et al*, 2007), a subsequent study has shown that in both mouse and human colorectal cancers, CD133 expression is not restricted to rare cell subsets, but it is detectable in a large majority of tumour cells, irrespective of their tumourigenicity (Shmelkov *et al*, 2008). Alternatively, the co-expression on tumour cells of CD44, CD166, and EpCAM molecules, has been reported to identify the CSC pool more precisely than CD133 expression alone (Dalerba *et al*, 2007b).

Despite the potentially high clinical relevance of CSCs, little is known about the prognostic value of the expression of putative CSC markers in colorectal cancers. Contradictory findings have been reported about the association between the expression of CD44, in particular of its v6 splicing variant, and tumour progression (Mulder *et al*, 1994; Herrlich *et al*, 1995; Weg-Remers *et al*, 1998). In a study based on 111 cases, membranous but not cytoplasmic expression of CD166 has been found to correlate with a shortened survival (Weichert *et al*, 2004). More recently, either a negative association or no correlation between high CD133 expression and clinical outcome has been reported in several independent studies including limited numbers of cases (Horst *et al*, 2008; Kojima *et al*, 2008; Choi *et al*, 2009; Li *et al*, 2009). Still missing is a comprehensive analysis of the expression of putative CSC markers in very large groups of patients, amenable to detailed statistical analysis. Moreover, the prognostic significance of the co-expression of multiple CSC markers within the same tumour has not been evaluated so far.

The aim of this study was to elucidate the expression and the prognostic role of CD133, CD166, CD44s, EpCAM, and ALDH1 expression in colorectal cancer, by using a tissue microarray including 1420 primary colorectal cancers with full clinicopathological data and follow-up. Results were further evaluated using 101 corresponding whole tissue sections and three established colorectal cancer cell lines.

## Materials and methods

### Patients and clinicopathological data

Archival paraffin-embedded material from 1420 patients with primary, pre-operatively untreated colorectal cancer were retrieved from multiple centres including the Institute of Pathology, University Hospital of Basel, Switzerland; the Institute of Clinical Pathology, Basel Switzerland; and the Institute of Pathology, Stadtspital Triemli, Zürich, Switzerland. All histopathological information was systematically re-reviewed from the corresponding hematoxylin and eosin slides including pT classification, pN classification, tumour grade, histological subtype, and the presence of vascular invasion. Tumour border configuration was diagnosed according to Jass *et al* (1987) as ‘pushing /expanding’ when there was a reasonably well-circumscribed margin at the invasive front and as ‘infiltrating’ when no recognisable margin of growth and a streaming dissection between normal structures of the bowel wall was present. Clinical information was retrieved from patient records and included age, gender, tumour location, and disease-specific survival time. For patients diagnosed at the Institute for Pathology, Stadtspital Triemli, Zürich, information on local recurrence (*n*=476), distant metastasis (*n*=489) and adjuvant therapy (*n*=478) was available. Patient characteristics are summarised in Table 1. The use of material in this study has been approved by the local ethics committee.

### Tissue microarray and immunohistochemistry

Tumour specimens from all 1420 patients as well as 57 samples of normal colonic mucosa were included on a previously described tissue microarray (Zlobec *et al*, 2009). Tissue cylinders with a diameter of 0.6 mm were punched from morphologically representative tissue areas of each ‘donor’ tissue block and brought into one recipient paraffin block (3 × 2.5 cm) using a homemade semiautomated tissue arrayer. Immunohistochemistry was performed for protein markers CD133, CD44s, CD166, EpCAM, and ALDH1. Detailed procedures have been described elsewhere (Zlobec *et al*, 2007a). The following primary antibodies were used: anti-human CD133 (clone C24B9; 1:100; Cell Signaling, Allschwil, Swizerland), anti-human CD166 (clone M0G/07; 1:200; Novocastra, Newcastle, UK), anti-human CD44s (clone DF1485; 1:50; Dako, Glostrup, Denmark), anti-human EpCAM (clone VU-1D9; 1:200; Novocastra), and anti-human ALDH1 isoform α1(polyclonal; 1:500; AbCam, Cambridge, UK). Negative controls underwent the same protocol with the primary antibody omitted.

### Evaluation of immunohistochemistry

**Figure 1:**
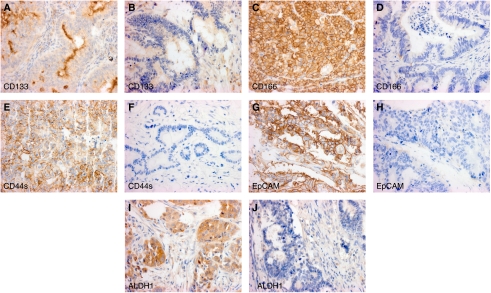
Colorectal cancer samples with membranous positivity and corresponding negative staining for CD133 (**A** and **B**), CD166 (**C** and **D**), CD44s (**E** and **F**), EpCAM (**G** and **H**) and cytoplasmic positivity and negativity for ALDH1 (**I** and **J**).

For CD133, CD166, CD44s, and EpCAM, only membranous staining was considered, whereas for ALDH1, cytoplasmic immunoreactivity was evaluated (Figure 1). Tissues were scored semi-quantitatively by evaluating the proportion of positive tumour cells over the total number of tumour cells (percentage of positive tumour cells per tissue microarray punch). Then, using receiver-operating characteristic (ROC) curve analysis (Zlobec *et al*, 2007b), appropriate cutoff scores for each marker were obtained. Positive staining in percentages of cells above or below the cutoff scores was classified as ‘overexpression’ or ‘loss’, respectively. The reliability of the cutoff score was obtained by 200 bootstrapped replications, a method which re-samples the data with replacement.

### Whole tissue sections

A total of 101 whole tissue sections from corresponding colorectal cancer patients included on the tissue microarray were retrieved and immunohistochemistry for the markers found to have prognostic value was performed according to the protocol outlined above. These cases were part of a previous study used to investigate the expression of putative stem cell markers within the regions of most dense tumour budding at the invasive front of colorectal cancers only (Hostettler *et al*, 2010). All slides were scored in the adjacent normal mucosa, if available, tumour centre, and at the invasive tumour front separately. Differences in expression pattern were described, namely, whether increased or decreased expression was observed from the normal adjacent tissue to the tumour centre and finally to the invasive tumour front.

### Tumour invasion assay

The colorectal cancer cell lines LS180, SW480, and Colo205 were cultured in RPMI 1640 medium supplemented, with GlutaMAX, MEM NEAA, 10 mM HEPES, 1 mM sodium pyruvate, kanamycin sulphate, and 10% FCS (all the reagents were from Gibco, Paisley, UK). For invasion assays, cells were stained with APC-conjugated anti-CD44s and PE-conjugated anti-CD166 antibodies (BD Pharmingen, San Jose, CA, USA), and CD44+/CD166+ or CD44−/CD166− cell subsets were sorted by flow cytometry. Dead cells were excluded by DAPI staining. Purity of sorted cells was ⩾97%. Unsorted tumour cells or sorted subsets were tested for invasiveness in a chemoinvasion assay (Albini and Benelli, 2007). Briefly, tumour cells re-suspended in serum-free medium were seeded in transwell plates on uncoated or matrigel-coated membranes (8 *μ*m pore size, BD Biocoat Tumour invasion assay, BD Biosciences, San Jose, CA, USA). Medium containing 5% FCS was seeded in the lower chambers and the cells were incubated at 37°C for 20 h. Inserts were then removed and numbers of cells migrated into the lower chambers were quantified by CyQUANT Cell Proliferation Assay Kit (Invitrogen, Paisley, UK). Percentages of cell invasion were calculated according to the following formula: (relative fluorescent units (RFU) of cells invaded through matrigel-coated membranes/mean RFU of cells migrated through uncoated membranes) × 100.

### Statistical analysis

The *χ*^2^-tests were carried out for categorical end points. The product-limit method and log-rank or Wilcoxon tests were used to assess differences in survival time. The 5-year survival rates and 95% confidence intervals (CI) were obtained. For Cox multiple regression analysis, the assumption of proportional hazards was verified before each analysis. Patients with missing clinicopathological data or with non-evaluable immunohistochemistry were excluded from the analysis. Hazard ratios (HR) and 95% CI were obtained to assess the prognostic effect of each protein marker on outcome. All tests were two-sided and *P*-values were considered statistically significant with *P*<0.05.

## Results

### Tissue microarray analysis

#### Normal mucosa *vs* colorectal cancer

The mean percentage of cells expressing CD133 was 0.5% in normal tissue and it significantly increased in colorectal tumours (24.7% *P*<0.001). Similarly, for CD44s and CD166, expression in normal tissue was detectable in 4.3 and 41.3% of cells on average, respectively, as compared with 33.1 and 64.4% in tumour (*P*<0.001). Percentages of EpCAM-expressing cells were slightly decreased in tumour compared with normal tissue, with a mean of 95.8 and 100% of positive cells, respectively (*P*<0.001). Similar results were noted for ALDH1, with an average expression of 15.1% in normal tissue as compared with 10.0% in tumour tissue (*P*<0.001).

### CD133

The CD133 expression was evaluated in 1245 cases. The cutoff score, based on ROC analysis, was fixed at 5%. Among the analysed cases, 616 cases (49.5%) displayed overexpression, whereas the remaining 629 cases (50.5%) showed loss. Neither overexpression nor loss of CD133 was significantly associated with tumour progression or survival time.

### CD166

**Figure 2:**
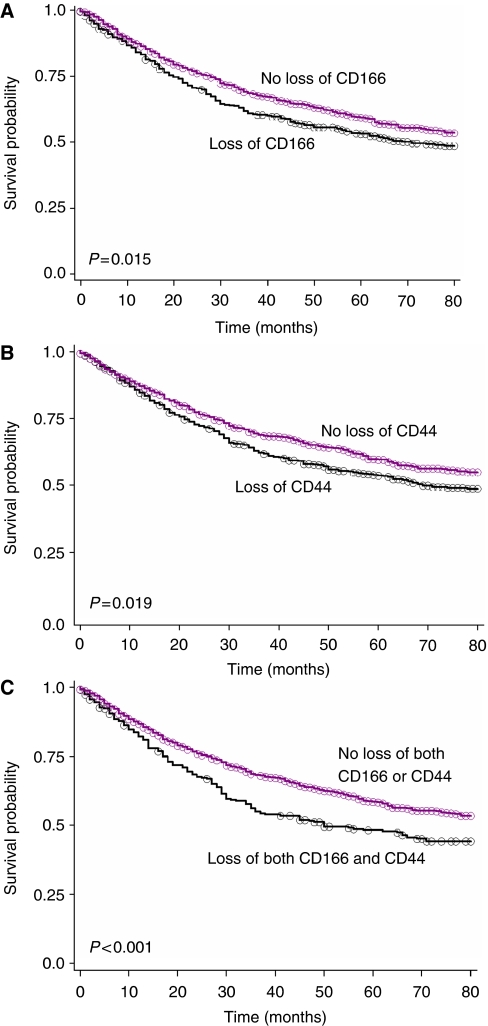
Kaplan–Meier survival curves illustrating survival time differences in patients with (**A**) loss *vs* overexpression of membranous CD166, (**B**) loss *vs* overexpression of CD44s, and (**C**) loss of both CD166 and CD44s *vs* all other combinations (loss of either CD166 or CD44s or none) on tissue microarray.

A total of 1274 cases were evaluable for CD166 (Table 2). Cutoff score was fixed at 65%, based on ROC analysis. Overexpression was detected in 775 cases (60.8%), whereas in the remaining 499 cases (39.2%), loss of expression was observed. Loss of membranous CD166 was linked to more advanced T classification (*P*=0.002), lymph node metastasis (*P*=0.004), an infiltrating tumour border configuration (*P*<0.001), and worse overall survival compared with patients with CD166 overexpression (*P*=0.015; Figure 2A). The 5-year survival rates were 52.9% (95% CI: 48–57%) and 59.0% (95% CI: 55–63%), respectively. In multivariable analysis including age, T classification, N classification, vascular invasion, tumour border configuration, and metastasis, loss of membranous CD166 did not show an independent adverse effect on survival. This was found again when analysed only with tumour border configuration, suggesting that the prognostic effect of loss of membranous CD166 in the tumour centre may be secondary to its association with these unfavourable prognostic features, in particular with the infiltrating tumour growth pattern.

### CD44s

CD44s expression could be evaluated on 1261 tumours and the cutoff score was fixed at 5% (Table 2). Of the analysed tumours, 607 (48.1%) showed loss and 654 (51.9%) overexpression. Loss of membranous CD44s was linked to more advanced T classification (*P*=0.014), lymph node involvement (*P*=0.002), the presence of vascular invasion (*P*=0.048), left-sidedness (*P*=0.008), and an infiltrating tumour border (*P*=0.002). Furthermore, in 467 cases, for which information on local recurrence was available, a trend between loss of CD44s and local recurrence (*P*=0.052) was also observed. The 5-year survival rate for patients with loss of membranous CD44s was 53.4% (95% CI: 49–58%), considerably poorer as compared with those with CD44s overexpression, which was 59.3% (95% CI: 55–63% *P*=0.019) (Figure 2B). However, the prognostic effect of CD44s was not independent of age, T classification, N classification, vascular invasion, the tumour border configuration, or metastasis. As seen for CD166, the absence of prognostic effect was again found when adjusting solely for the tumour border configuration. These results seem to indicate that the unfavourable prognostic impact of loss of membranous CD44s within the main tumour body may be secondary to its association with T classification, lymph node metastasis, the presence of vascular invasion, and the infiltrating growth pattern.

### EpCAM

Membranous EpCAM expression was evaluable in 1278 cases of which 1145 (89.6%) showed a diffuse staining in 100% of tumour cells (Table 2). A total of 133 tumours (10.4%) showed loss of membranous EpCAM (<100% staining, as defined according to ROC analysis) and were significantly associated with the presence of lymph node metastasis (*P*=0.023) and an infiltrating tumour margin (*P*=0.005), as well as with a trend towards higher tumour grade (*P*=0.088) and the presence of vascular invasion (*P*=0.056). Furthermore, in 489 cases, a decreased EpCAM expression showed a trend towards the presence of distant metastasis (*P*=0.084). No association of EpCAM expression with survival time was noted either in univariate analysis or after adjusting for the effect of the tumour border configuration.

### ALDH1

Of the 1287 tumours evaluable for cytoplasmic ALDH1 expression, 987 (76.7%) had 0% immunoreactive tumour cells. Overexpression of ALDH1 expression, defined on the basis of ROC analysis as >25% of positive cells, which was observed in the remaining 300 cases (23.3%), was related to higher tumour grade (*P*=0.025) but not to differences in survival time.

### Multi-marker combination CD166/CD44s

Cancer stem cells derived from colorectal cancer have been reported to co-express several surface markers, in particular CD166, CD44s, and EpCAM molecules (Dalerba *et al*, 2007b). It was therefore interesting for us to evaluate clinical relevance of simultaneous loss or overexpression of these markers in colorectal cancer tissues. However, as EpCAM expression was very high in all cases (mean expression 95.8%) and it did not correlate with survival time, we mainly focused on co-expression of CD166 and CD44s molecules. Concomitant loss of CD166 and CD44s molecules was observed in 276 tumours, whereas the remaining 884 cases displayed various combinations of these two markers including 275 CD166+/CD44s− cases, 180 CD166−/CD44s+ cases, and 429 double-positive cases. Importantly, the 5-year survival rate for cases displaying loss of both CD166 and CD44s expression was 48.3% (95% CI: 42–54%), whereas that for the remaining multi-marker phenotypes was 58.6% (95% CI: 55–62% *P*<0.001) (Figure 2C). Despite this adverse effect and similarity to each marker alone, the prognostic value of this combination of CD166 and CD44s did not contribute independent prognostic information in multivariable analysis, indicating no added benefit in risk stratification for patients with colorectal cancer in this series when evaluating double negativity compared with single negativity and effect on outcome.

### Whole tissue sections

**Figure 3:**
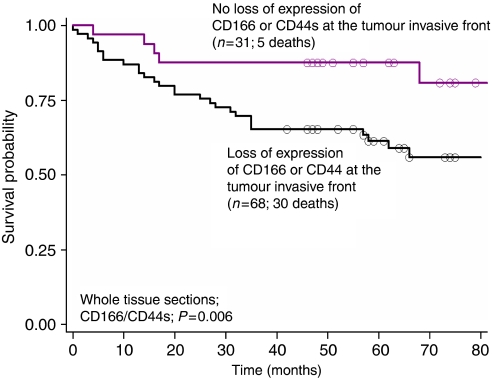
Kaplan–Meier survival curves illustrating survival time differences in patients with loss of both CD166 and CD44s *vs* all other combinations (loss of either CD166 or CD44s or none) on whole tissue sections.

The above findings suggested a positive association between loss of CD166 and CD44s molecules and more unfavourable prognosis. In view of the fact that surface molecules might be heterogeneously expressed within colorectal cancer tissues (Haier *et al*, 2000), CD166 and CD44s staining was further evaluated on whole tissue sections from 101 patients previously included on this tissue microarray (Hostettler *et al*, 2010). We analysed first protein expression in the tumour centre as compared with normal adjacent mucosa. Indeed, increased expression of CD166 and CD44s in the tumour centre compared with normal mucosa occurred in 65 of 89 (73% *P*<0.001) and 56 of 89 (62.9% *P*=0.008) cases, respectively, thus confirming our tissue microarray findings. Next, we compared protein expression in the tumour centre with that at the tumour invasive front. Our results indeed showed a heterogeneous expression pattern for both CD166 and CD44s. In particular, decreased expression from the tumour centre to the tumour border was observed in 51 of 100 cases for CD166 and in 47 of 99 cases for CD44s. Importantly, 80.4% (*P*<0.001) of cases showing reduced expression of CD166 and 78% (*P*<0.001) of those showing reduced expression of CD44s had an infiltrating border configuration, thus, confirming a positive association between loss of CD166 or CD44s and tumour spreading. Third, we evaluated whether this expression pattern resulted in a poorer effect on survival. Patients with tumours showing decreased levels of CD166 or CD44s expression towards the invasive tumour front when compared with the tumour centre had a significantly more adverse outcome compared with those with no loss of either marker (*P*=0.006) (Figure 3). Notably, this result was maintained in multivariable analysis with the tumour border configuration. In particular, HR (95% CI) for the combined analysis of CD166/CD44s and tumour border configuration were 4.32 (1.3–14.3; *P*=0.017) and 1.73 (0.7–4.3; *P*=0.232), respectively, indicating that the poorer outcome in patients with an expression pattern showing a loss of CD166 and CD44s expression towards the invasive front, although highly linked to tumour growth pattern, may be independent of this histological parameter. Thus, despite the possible heterogeneity between expression levels in the tumour centre and tumour front, a diminished expression of CD166 and CD44s seemed to be consistently associated with tumour progression and unfavourable clinical outcome.

### Invasiveness of tumour cells differing in CD44s and CD166 expression

**Figure 4:**
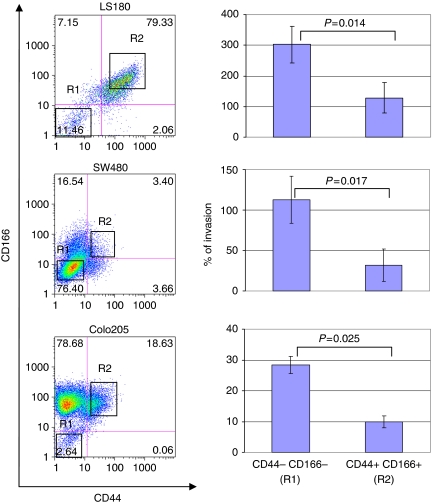
The CD44−/CD166− tumour cells display higher invasive potential than CD44+/CD166+ cells. The CD44−/CD166− and CD44+/CD166+ cell subsets were sorted by flow cytometry, according to the gates depicted, from LS180, SW480, and Colo205 cell lines. Sorted subsets were tested in invasion assays. Percentages of cell invasion (mean values±s.d.) are shown. Data are representative of six independent experiments.

As CD44s and CD166 are adhesion molecules, we hypothesised that their loss might directly favour the invasiveness of tumour cells, possibly as a consequence of reduced adhesion (Figure 4). To address this issue in a controlled ‘*in vitro*’ model, we investigated the invasive potential of CD44+/CD166+ or CD44−/CD166− cells derived from the human colorectal cancer cell lines, LS180, SW480, and Colo205. All three cell lines displayed a heterogeneous surface expression of CD44 and CD166 (Figure 4, left panels). However, when CD44+/CD166+ and CD44−/CD166− cell subsets were sorted and evaluated for their invasive capacity, in all cases, the double-negative fractions exhibited significantly higher invasive potential than their positive counterparts (Figure 4, right panels). These results suggest that absence of CD44 and CD166 molecules is directly associated with higher invasive capacity of tumour cells.

## Discussion

In this study, we have evaluated the relationship between expression of five putative CSC markers and the most clinically relevant features of colorectal cancer. Our findings suggest that, despite the increased expression of some of these markers, including CD133, CD166, and CD44s, from normal to early colorectal cancer, it is the overall decreased membranous expression, particularly of EpCAM, CD166, and CD44s, which is linked to a more aggressive tumour phenotype.

The CD44s has long been thought of as a marker of tumour invasiveness and metastasis and recently has also been described as a putative colorectal CSC marker (Visvader and Lindeman, 2008). Many early works investigating the CD44s gene and its splice variants report a poorer impact on survival time in patients with increased expression levels of the gene or protein (Mulder *et al*, 1994; Wielenga *et al*, 1998). However, more recent results are far from unanimous, suggesting either no role for CD44s (or variant isoforms) or a worse clinical outcome with loss of protein expression (Coppola *et al*, 1998; Morrin and Delaney, 2002; Ngan *et al*, 2007; Choi *et al*, 2009; Huh *et al*, 2009). Others describe an increased expression of CD44s from normal to adenoma to carcinoma, a finding that is in line with the results of this study including normal and tumour tissue (Coppola *et al*, 1998; Weg-Remers *et al*, 1998). Relatively fewer studies have evaluated the prognostic impact of CD166 in colorectal cancer. Weichert *et al* (2004) described increased expression of CD166 from normal to tumour tissue, and, in a group of 111 colorectal cancer cases, observed correlation between membranous, but not cytoplasmic, CD166 expression and shortened survival. Patel *et al* (2009) also found a significant increase in CD166 expression in adenomatous glands and an age-dependent increase in CD44s and CD166 expression, correlating further with the number of polyps. Their findings suggest a role for CD44s and CD166 in tumour development from the pre-cancerous state.

Although, in this study, we confirm the increased expression of both CD44s and CD166 from normal adjacent colorectal tissue to cancer, our results support the association of loss (rather than increase) of membranous CD44s and CD166 with aggressive tumour-related features such as more advanced pT stage, pN stage, vascular invasion, and an infiltrating tumour growth pattern. In addition, we document a poorer survival time with loss of membranous expression of both these protein markers in univariate but not multivariable survival time analysis, indicating that the poor prognostic impact of CD44s and CD166 may be secondary to their association with other established prognostic criteria. A similar result has been reported in other tumour types, including ovarian and prostate cancer (Kristiansen *et al*, 2003; Mezzanzanica *et al*, 2008). Expression of EpCAM has previously been linked to poorer survival time in several tumour types including breast cancer (Gastl *et al*, 2000), gallbladder tumours (Prince *et al*, 2008), and those of the Papilla Vateri (Scheunemann *et al*, 2007; Prince *et al*, 2008). On investigating rectal cancers, Gosens *et al* (2007) found strong membranous EpCAM staining in the tumour centre and a progressive loss at the tumour front associated with high tumour grade, tumour budding, and a poor local and distant recurrence-free survival. In the present study, the decreased EpCAM expression was also found to be significantly linked to features of tumour invasion, including presence of lymph node metastasis and infiltrating tumour margin, and it showed a trend with higher tumour grade, presence of vascular invasion, and presence of distant metastasis. Altogether, these studies suggest that diminished EpCAM expression is related to tumour invasiveness and progression.

We hypothesise that our findings concerning decreased (rather than increased) expression of membranous CD166, CD44s, and EpCAM and their association with features of tumour progression are in large part a result of their cell adhesion function. Loss of cell adhesion is known to be a fundamental mechanism underlying the initiation of the metastatic process (Woodhouse *et al*, 1997). In fact, decreased expression of other cell adhesion molecules such as E-cadherin and CD44v6, are lost at the invasive front of colorectal cancer (Ngan *et al*, 2007; Zlobec *et al*, 2007a). Moreover, loss of E-cadherin expression is highlighted as a key event in epithelial–mesenchymal transition (EMT) (Brabletz *et al*, 2005a). In colorectal cancer, EMT-derived tumour cells are histologically represented by the presence of ‘tumour budding’ at the invasive front and are almost always present in tumours with an infiltrating tumour growth pattern. Tumour budding cells are defined as single cells or small clusters of de-differentiated tumour cells (Prall, 2007) and are thought to represent migratory stem cells (Brabletz *et al*, 2005b). High numbers of tumour budding cells are recognised as independent and adverse prognostic features as their presence is predictive of vascular and lymphatic invasion (Prall, 2007). In our previous study directly focusing on the expression of putative stem cell markers in EMT-derived tumour cells, we describe a low frequency of CD44s, CD166, and EpCAM staining within tumour buds, thus emphasising the loss of cell adhesion molecules typical of these cells (Hostettler *et al*, 2010). Here, we find that even within representative regions of the main tumour body obtained using TMA analysis, loss of CD44s, CD166, and EpCAM is associated with more aggressive tumour-related features and not surprisingly with an infiltrating growth pattern, an observation which is directly in line with the low frequency found within tumour buds. Moreover, using whole tissue sections, we report the same dynamic and findings, namely, the correlation between loss of expression of CD44s and CD166 towards the invasive front and poorer clinical outcome. Our results support the notion that the evaluation of membranous CD44s, CD166, and EpCAM expression assessed by immunohistochemistry may be representative of their cell adhesion function.

We could not confirm the prognostic value of CD133 or ALDH1 in this study (Ginestier and Wicha, 2007; Horst *et al*, 2008, 2009; Kojima *et al*, 2008; Choi *et al*, 2009; Huang *et al*, 2009). Several reasons for these discrepancies can be hypothesised including differences in sample size (power for detecting prognostic differences), methodology (tissue microarray *vs* whole tissue sections), and certainly the choice of cutoff scores for the definition of positive staining or staining intensity. Moreover, the intra-cellular localisation of the evaluated staining (membranous/cytoplasmic) must also be discussed. For example, although EpCAM, similar to CD44, is known for its cell adhesion function (membranous localisation), it seems to have versatile roles in signalling, cell migration, proliferation, and differentiation, depending on the microenvironment (cytoplasmic localisation) (Trzpis *et al*, 2007).

A few factors might be envisaged as potential limitations of our study. First, information on local recurrence, distant metastasis, and post-operative therapy was only available for patients treated at one diagnostic centre. However, the lack of independent prognostic effects for our two main CSC markers of interest, namely, CD166 and CD44s, suggest that the absence of complete treatment information may only minimally influence our findings. The results of this study also highlight a heterogeneous expression of CD166 and CD44s throughout the tumour. These findings suggest that using single-punch tissue microarray analysis to investigate these and likely other cell adhesion molecules may be suboptimal. Nonetheless, using two additional and different approaches, namely, analysis of whole tissues sections and *in vitro* analysis using three cell lines, we could show similar findings. Although established cell lines might not fully reproduce the behaviour of primary tumours, our *in vitro* findings strongly suggest that CD44s and CD166 are of functional importance in limiting tumour cell spreading in surrounding tissues, thus underlining the hypothesis that loss of expression of these markers, rather than their overexpression, is associated with a more aggressive tumour phenotype.

To our knowledge, this is the first systematic assessment of the prognostic value of CD133, CD166, CD44, EpCAM, and ALDH1 in colorectal tumours evaluated on a large number of cases. Our findings indicate that expression of CSC markers is not *per se* predictive of poor clinical outcome. Loss of expression of CD166, CD44s, and EpCAM is rather linked to an aggressive tumour phenotype, particularly, to the presence of an infiltrating tumour margin that may implicate these proteins and their loss of membranous expression in events occurring at the invasive tumour front.

## Figures and Tables

**Table 1:** Summary of patient characteristics (*n*=1420)

**Clinicopathological feature**	**Outcome**	**Frequency *N* (%)**
Age (years; *n*=1420)	Mean (range)	69.9 (30–96)
Gender (*n*=1414)	Female	741 (52.4)
	Male	673 (47.6)
		
Histological subtype (*n*=1420)	Mucinous	119 (8.4)
	Other	1301 (91.6)
		
Tumour location (*n*=1400)	Right sided	488 (34.9)
	Left sided	430 (30.7)
	Rectum	482 (34.4)
		
T classification (*n*=1387)	pT1	62 (4.5)
	pT2	203 (14.6)
	pT3	899 (64.8)
	pT4	223 (16.1)
		
N classification (*n*=1363)	N0	711 (52.2)
	N1	358 (26.3)
	N2	294 (21.6)
		
Tumour grade (*n*=1385)	G1	31 (2.2)
	G2	1177 (85.0)
	G3	177 (12.8)
		
Vascular invasion (*n*=1385)	Absent	1002 (72.4)
	Present	383 (27.7)
		
Tumour border configuration	Infiltrating	871 (62.9)
(*n*=1384)	Pushing	513 (37.1)
		
Local recurrence (*n*=476)	Absent	276 (58.0)
	Present	200 (42.0)
		
Distant metastasis (*n*=489)	Absent	401 (82.0)
	Present	88 (18.0)
		
Post-operative therapy (*n*=478)	No	377 (78.9)
	Yes	101 (21.1)
		
Survival time (months) (*n*=1379)	5-year survival rate	56.4 (54–59)

**Table 2:** Association of membranous CD166, CD44s, and EpCAM with clinicopathological features in colorectal cancer patients.

	**CD166, *N* (%)**			**CD44s, *N* (%)**			**EpCAM, *N* (%)**		
**Clinicopathological feature**	**Loss**	**Overexpression**	***P*-value**	**Loss**	**Overexpression**	***P*-value**	**Loss**	**Overexpression**	***P*-value**
*T classification*									
pT1–2	72 (14.7)	165 (21.7)	0.002	96 (16.0)	137 (21.5)	0.014	23 (18.1)	214 (19.0)	0.814
pT3–4	417 (85.3)	594 (78.3)		503 (84.0)	500 (78.5)		104 (81.9)	914 (81.0)	
									
*N classification*									
pN0	228 (47.4)	417 (55.9)	0.004	275 (47.2)	353 (56.0)	0.002	55 (43.0)	594 (53.6)	0.023
pN1–2	253 (52.6)	329 (44.1)		308 (52.8)	277 (44.0)		73 (57.0)	515 (46.4)	
									
*Tumour grade*									
G1–2	438 (89.9)	658 (86.8)	0.097	611 (88.7)	474 (87.0)	0.361	103 (82.4)	990 (87.8)	0.088
G3	49 (10.1)	100 (13.2)		78 (11.3)	71 (11.0)		22 (17.6)	138 (12.2)	
									
*Vascular invasion*									
Absent	340 (69.8)	564 (74.4)	0.076	418 (69.6)	473 (74.6)	0.048	82 (65.1)	824 (73.1)	0.056
Present	147 (30.2)	194 (25.6)		183 (30.4)	161 (25.4)		44 (34.9)	303 (26.9)	
									
*Tumour border configuration*									
Pushing	140 (28.8)	323 (42.6)	<0.001	197 (32.8)	261 (41.3)	0.002	32 (25.4)	429 (38.1)	0.005
Infiltrating	346 (71.2)	435 (57.4)		404 (67.2)	371 (58.7)		94 (74.6)	698 (61.9)	
									
*Tumour location*									
Left sided	328 (66.1)	492 (64.4)	0.529	476 (68.7)	341 (61.4)	0.008	89 (67.4)	737 (65.1)	0.596
Right sided	168 (33.9)	272 (35.6)		217 (31.3)	214 (38.6)		43 (32.6)	395 (64.9)	
									
*Local recurrence*									
Absent	43 (50.6)	201 (60.2)	0.109	133 (54.7)	121 (64.0)	0.052	18 (54.6)	239 (57.7)	0.722
Present	42 (49.4)	133 (39.8)		110 (45.3)	68 (36.0)		15 (45.5)	175 (42.3)	
									
*Metastasis*									
Absent	70 (79.6)	275 (81.4)	0.699	202 (82.5)	159 (82.0)	0.894	23 (69.7)	345 (82.0)	0.084
Present	18 (20.5)	63 (18.6)		43 (17.6)	35 (18.0)		10 (30.3)	76 (18.1)	
									
*Survival rate (95*% *CI)*									
5 year	52.9 (48-57)	59.0 (55-63)	0.015	53.4 (49-58)	59.3 (55-63)	0.019	54.6 (45-63)	56.6 (53-60)	0.521

Abbreviations: CI=confidence interval; ROC=receiver-operating characteristic.

Cutoff scores for overexpression derived from ROC curve analysis were 65% for CD166, 5% for CD44s, and 100% for EpCAM.

## References

[bib1] Albini A, Benelli R (2007) The chemoinvasion assay: a method to assess tumor and endothelial cell invasion and its modulation. Nat Protoc 2: 504–511 17406614 10.1038/nprot.2006.466

[bib2] Brabletz T, Hlubek F, Spaderna S, Schmalhofer O, Hiendlmeyer E, Jung A, Kirchner T (2005a) Invasion and metastasis in colorectal cancer: epithelial-mesenchymal transition, mesenchymal-epithelial transition, stem cells and beta-catenin. Cells Tissues Organs 179: 56–65 15942193 10.1159/000084509

[bib3] Brabletz T, Jung A, Spaderna S, Hlubek F, Kirchner T (2005b) Opinion: migrating cancer stem cells - an integrated concept of malignant tumour progression. Nat Rev Cancer 5: 744–749 16148886 10.1038/nrc1694

[bib4] Choi D, Lee HW, Hur KY, Kim JJ, Park GS, Jang SH, Song YS, Jang KS, Paik SS (2009) Cancer stem cell markers CD133 and CD24 correlate with invasiveness and differentiation in colorectal adenocarcinoma. World J Gastroenterol 15: 2258–2264 19437567 10.3748/wjg.15.2258 PMC2682242

[bib5] Coppola D, Hyacinthe M, Fu L, Cantor AB, Karl R, Marcet J, Cooper DL, Nicosia SV, Cooper HS (1998) CD44V6 expression in human colorectal carcinoma. Hum Pathol 29: 627–635 9635685 10.1016/s0046-8177(98)80014-2

[bib6] Dalerba P, Cho RW, Clarke MF (2007a) Cancer stem cells: models and concepts. Annu Rev Med 58: 267–284 17002552 10.1146/annurev.med.58.062105.204854

[bib7] Dalerba P, Dylla SJ, Park IK, Liu R, Wang X, Cho RW, Hoey T, Gurney A, Huang EH, Simeone DM, Shelton AA, Parmiani G, Castelli C, Clarke MF (2007b) Phenotypic characterization of human colorectal cancer stem cells. Proc Natl Acad Sci USA 104: 10158–10163 17548814 10.1073/pnas.0703478104 PMC1891215

[bib8] Dean M, Fojo T, Bates S (2005) Tumour stem cells and drug resistance. Nat Rev Cancer 5: 275–284 15803154 10.1038/nrc1590

[bib9] Gastl G, Spizzo G, Obrist P, Dunser M, Mikuz G (2000) Ep-CAM overexpression in breast cancer as a predictor of survival. Lancet 356: 1981–1982 11130529 10.1016/S0140-6736(00)03312-2

[bib10] Ginestier C, Wicha MS (2007) Mammary stem cell number as a determinate of breast cancer risk. Breast Cancer Res 9: 109 17688678 10.1186/bcr1741 PMC2206714

[bib11] Gosens MJ, van Kempen LC, van de Velde CJ, van Krieken JH, Nagtegaal ID (2007) Loss of membranous Ep-CAM in budding colorectal carcinoma cells. Mod Pathol 20: 221–232 17361206 10.1038/modpathol.3800733

[bib12] Herrlich P, Pals S, Ponta H (1995) CD44 in colon cancer. Eur J Cancer 31A: 1110–1112 7577002 10.1016/0959-8049(95)00252-e

[bib13] Horst D, Kriegl L, Engel J, Kirchner T, Jung A (2008) CD133 expression is an independent prognostic marker for low survival in colorectal cancer. Br J Cancer 99: 1285–1289 18781171 10.1038/sj.bjc.6604664 PMC2570510

[bib14] Horst D, Kriegl L, Engel J, Kirchner T, Jung A (2009) Prognostic significance of the cancer stem cell markers cd133, cd44, and cd166 in colorectal cancer. Cancer Invest 27(8): 844–850 19626493 10.1080/07357900902744502

[bib15] Hostettler L, Zlobec I, Terracciano L, Lugli A (2010) ABCG5-positivity in tumor buds is an indicator of poor prognosis in node-negative colorectal cancer patients. World J Gastroenterol 16: 732–739 20135722 10.3748/wjg.v16.i6.732 PMC2817062

[bib16] Huang EH, Hynes MJ, Zhang T, Ginestier C, Dontu G, Appelman H, Fields JZ, Wicha MS, Boman BM (2009) Aldehyde dehydrogenase 1 is a marker for normal and malignant human colonic stem cells (SC) and tracks SC overpopulation during colon tumorigenesis. Cancer Res 69: 3382–3389 19336570 10.1158/0008-5472.CAN-08-4418 PMC2789401

[bib17] Huh JW, Kim HR, Kim YJ, Lee JH, Park YS, Cho SH, Joo JK (2009) Expression of standard CD44 in human colorectal carcinoma: association with prognosis. Pathol Int 59: 241–246 19351367 10.1111/j.1440-1827.2009.02357.x

[bib18] Jass JR, Love SB, Northover JM (1987) A new prognostic classification of rectal cancer. Lancet 1: 1303–1306 2884421 10.1016/s0140-6736(87)90552-6

[bib19] Kojima M, Ishii G, Atsumi N, Fujii S, Saito N, Ochiai A (2008) Immunohistochemical detection of CD133 expression in colorectal cancer: a clinicopathological study. Cancer Sci 99: 1578–1583 18754869 10.1111/j.1349-7006.2008.00849.x PMC11159232

[bib20] Kristiansen G, Pilarsky C, Wissmann C, Stephan C, Weissbach L, Loy V, Loening S, Dietel M, Rosenthal A (2003) ALCAM/CD166 is up-regulated in low-grade prostate cancer and progressively lost in high-grade lesions. Prostate 54: 34–43 12481253 10.1002/pros.10161

[bib21] Li CY, Li BX, Liang Y, Peng RQ, Ding Y, Xu DZ, Zhang X, Pan ZZ, Wan DS, Zeng YX, Zhu XF, Zhang XS (2009) Higher percentage of CD133+ cells is associated with poor prognosis in colon carcinoma patients with stage IIIB. J Transl Med 7: 56 19583834 10.1186/1479-5876-7-56 PMC2715381

[bib22] Mezzanzanica D, Fabbi M, Bagnoli M, Staurengo S, Losa M, Balladore E, Alberti P, Lusa L, Ditto A, Ferrini S, Pierotti MA, Barbareschi M, Pilotti S, Canevari S (2008) Subcellular localization of activated leukocyte cell adhesion molecule is a molecular predictor of survival in ovarian carcinoma patients. Clin Cancer Res 14: 1726–1733 18347173 10.1158/1078-0432.CCR-07-0428

[bib23] Morrin M, Delaney PV (2002) CD44v6 is not relevant in colorectal tumour progression. Int J Colorectal Dis 17: 30–36 12018451 10.1007/s003840100335

[bib24] Mulder JW, Kruyt PM, Sewnath M, Oosting J, Seldenrijk CA, Weidema WF, Offerhaus GJ, Pals ST (1994) Colorectal cancer prognosis and expression of exon-v6-containing CD44 proteins. Lancet 344: 1470–1472 7526103 10.1016/s0140-6736(94)90290-9

[bib25] Ngan CY, Yamamoto H, Seshimo I, Ezumi K, Terayama M, Hemmi H, Takemasa I, Ikeda M, Sekimoto M, Monden M (2007) A multivariate analysis of adhesion molecules expression in assessment of colorectal cancer. J Surg Oncol 95: 652–662 17443723 10.1002/jso.20638

[bib26] O’Brien CA, Pollett A, Gallinger S, Dick JE (2007) A human colon cancer cell capable of initiating tumour growth in immunodeficient mice. Nature 445: 106–110 17122772 10.1038/nature05372

[bib27] Pardal R, Clarke MF, Morrison SJ (2003) Applying the principles of stem-cell biology to cancer. Nat Rev Cancer 3: 895–902 14737120 10.1038/nrc1232

[bib28] Patel BB, Yu Y, Du J, Levi E, Phillip PA, Majumdar AP (2009) Age-related increase in colorectal cancer stem cells in macroscopically normal mucosa of patients with adenomas: a risk factor for colon cancer. Biochem Biophys Res Commun 378: 344–347 19010307 10.1016/j.bbrc.2008.10.179 PMC2644999

[bib29] Prall F (2007) Tumour budding in colorectal carcinoma. Histopathology 50: 151–162 17204028 10.1111/j.1365-2559.2006.02551.x

[bib30] Prince S, Zeidman A, Dekel Y, Ram E, Koren R (2008) Expression of epithelial cell adhesion molecule in gallbladder carcinoma and its correlation with clinicopathologic variables. Am J Clin Pathol 129: 424–429 18285265 10.1309/H8JEEAEB69J3KYND

[bib31] Reya T, Morrison SJ, Clarke MF, Weissman IL (2001) Stem cells, cancer, and cancer stem cells. Nature 414: 105–111 11689955 10.1038/35102167

[bib32] Ricci-Vitiani L, Lombardi DG, Pilozzi E, Biffoni M, Todaro M, Peschle C, De Maria R (2007) Identification and expansion of human colon-cancer-initiating cells. Nature 445: 111–115 17122771 10.1038/nature05384

[bib33] Scheunemann P, Stoecklein NH, Rehders A, Bidde M, Metz S, Peiper M, Eisenberger C, Schulte Am Esch J, Knoefel WT, Hosch SB (2007) Frequency and prognostic significance of occult tumor cells in lymph nodes in patients with adenocarcinoma of the papilla of Vater. HPB (Oxford) 9: 135–139 18333129 10.1080/13651820601090646 PMC2020782

[bib34] Shmelkov SV, Butler JM, Hooper AT, Hormigo A, Kushner J, Milde T, St Clair R, Baljevic M, White I, Jin DK, Chadburn A, Murphy AJ, Valenzuela DM, Gale NW, Thurston G, Yancopoulos GD, D’Angelica M, Kemeny N, Lyden D, Rafii S (2008) CD133 expression is not restricted to stem cells, and both CD133+ and CD133- metastatic colon cancer cells initiate tumors. J Clin Invest 118: 2111–2120 18497886 10.1172/JCI34401 PMC2391278

[bib35] Trzpis M, McLaughlin PM, de Leij LM, Harmsen MC (2007) Epithelial cell adhesion molecule: more than a carcinoma marker and adhesion molecule. Am J Pathol 171: 386–395 17600130 10.2353/ajpath.2007.070152 PMC1934518

[bib36] Visvader JE, Lindeman GJ (2008) Cancer stem cells in solid tumours: accumulating evidence and unresolved questions. Nat Rev Cancer 8: 755–768 18784658 10.1038/nrc2499

[bib37] Weg-Remers S, Anders M, von Lampe B, Riecken EO, Schuder G, Feifel G, Zeitz M, Stallmach A (1998) Decreased expression of CD44 splicing variants in advanced colorectal carcinomas. Eur J Cancer 34: 1607–1611 9893637 10.1016/s0959-8049(98)00177-4

[bib38] Weichert W, Knosel T, Bellach J, Dietel M, Kristiansen G (2004) ALCAM/CD166 is overexpressed in colorectal carcinoma and correlates with shortened patient survival. J Clin Pathol 57: 1160–1164 15509676 10.1136/jcp.2004.016238 PMC1770486

[bib39] Wielenga VJ, van der Voort R, Mulder JW, Kruyt PM, Weidema WF, Oosting J, Seldenrijk CA, van Krimpen C, Offerhaus GJ, Pals ST (1998) CD44 splice variants as prognostic markers in colorectal cancer. Scand J Gastroenterol 33: 82–87 9489913 10.1080/00365529850166257

[bib40] Woodhouse EC, Chuaqui RF, Liotta LA (1997) General mechanisms of metastasis. Cancer 80: 1529–1537 9362419 10.1002/(sici)1097-0142(19971015)80:8+<1529::aid-cncr2>3.3.co;2-#

[bib41] Zhou BB, Zhang H, Damelin M, Geles KG, Grindley JC, Dirks PB (2009) Tumour-initiating cells: challenges and opportunities for anticancer drug discovery. Nat Rev Drug Discov 8: 806–823 19794444 10.1038/nrd2137

[bib42] Zlobec I, Gunthert U, Tornillo L, Iezzi G, Baumhoer D, Terracciano L, Lugli A (2009) Systematic assessment of the prognostic impact of membranous CD44v6 protein expression in colorectal cancer. Histopathology 55: 564–575 19912362 10.1111/j.1365-2559.2009.03421.x

[bib43] Zlobec I, Lugli A, Baker K, Roth S, Minoo P, Hayashi S, Terracciano L, Jass JR (2007a) Role of APAF-1, E-cadherin and peritumoral lymphocytic infiltration in tumour budding in colorectal cancer. J Pathol 212: 260–268 17516584 10.1002/path.2164

[bib44] Zlobec I, Steele R, Terracciano L, Jass JR, Lugli A (2007b) Selecting immunohistochemical cut-off scores for novel biomarkers of progression and survival in colorectal cancer. J Clin Pathol 60: 1112–1116 17182662 10.1136/jcp.2006.044537 PMC2014838

[bib45] Haier J, Nasralla M, Nicolson GL 2000 Cell surface molecules and their prognostic values in assessing colorectal carcinomas. Ann Surg 231: 11–24 10636097 10.1097/00000658-200001000-00003 PMC1420960

